# Numerosity as a visual property: Evidence from two highly evolutionary distant species

**DOI:** 10.3389/fphys.2023.1086213

**Published:** 2023-02-09

**Authors:** Mercedes Bengochea, Bassem Hassan

**Affiliations:** Institut du Cerveau-Paris Brain Institute (ICM), Sorbonne Université, Inserm, CNRS, Hôpital Pitié-Salpêtrière, Paris, France

**Keywords:** numerosity, fruit flies, neural circuit, visual system, humans

## Abstract

Most animals, from humans to invertebrates, possess an ability to estimate numbers. This evolutionary advantage facilitates animals’ choice of environments with more food sources, more conspecifics to increase mating success, and/or reduced predation risk among others. However, how the brain processes numerical information remains largely unknown. There are currently two lines of research interested in how numerosity of visual objects is perceived and analyzed in the brain. The first argues that numerosity is an advanced cognitive ability processed in high-order brain areas, while the second proposes that “numbers” are attributes of the visual scene and thus numerosity is processed in the visual sensory system. Recent evidence points to a sensory involvement in estimating magnitudes. In this Perspective, we highlight this evidence in two highly evolutionary distant species: humans and flies. We also discuss the advantages of studying numerical processing in fruit flies in order to dissect the neural circuits involved in and required for numerical processing. Based on experimental manipulation and the fly connectome, we propose a plausible neural network for number sense in invertebrates.

## Introduction

An understanding of numbers is often interpreted as a distinctly human capacity and hallmark of our intelligence that, along with language, sets us apart from other animals. However many if not most animal species possess the ability to rapidly estimate numbers (Butterworth et al., 2017). Numerical cognition is a fundamental skill that is essential for an animal’s everyday life. For example, the evolutionary advantage of this skill is to facilitate animals’ choices of niches with more food, adequate interaction with conspecifics and fewer competitors thereby avoiding predation risk. Members of all vertebrate classes present numerical competence (Nieder, 2020). However, vertebrates are not the only ones. It has been shown that invertebrates are also endowed with numerical skills (Bortot et al., 2021) demonstrating that large brains are not a prerequisite for numerical cognition.

How numerical information is perceived and processed in the brain is a major question in the field of cognitive neuroscience. Numerosity is generally interpreted with two different approaches: as a highly abstract cognitive property or as a high-level visual feature. The first approach understands that numerosity is a concept that can be referred to by stimuli in different modalities and presentation modes, concretely or *via* symbols. Like this, numerical cognition relies on interactions of distinct functional circuits between multiple brain areas, including those supporting working memory and quantity processing (Menon, 2016). The second approach interprets that numerosity is a property computed by one given perceptual modality, such as the number of objects in a visual image. It has been shown that non-human animals [see (Nieder, 2020; Bortot et al., 2021; Lorenzi et al., 2021) for reviews] as well as newborn humans can instantaneously perceive numerical information from a scene without needing to count the amount of elements (Izard et al., 2009), suggesting the existence of a basic numerosity processing. Similarly, increasing evidence indicates that numerosity itself is represented and perceived as a visual feature in adult humans (Burr and Ross, 2008) pointing to an sensory processing of the numerical information. Accordingly, we recently showed a requirement for visual neurons in the third optic neuropil of fruit flies in spontaneous numerical processing, but not higher central brain areas previously predicted to be involved (Giurfa, 2019). These neurons connect processing of the retinal output in the optic lobe with central brain structures suggesting that, similarly to adult humans- *Drosophila melanogaster* also encodes visual numerosity as a sensory feature.

## Numerical computation in humans

**FIGURE 1:**
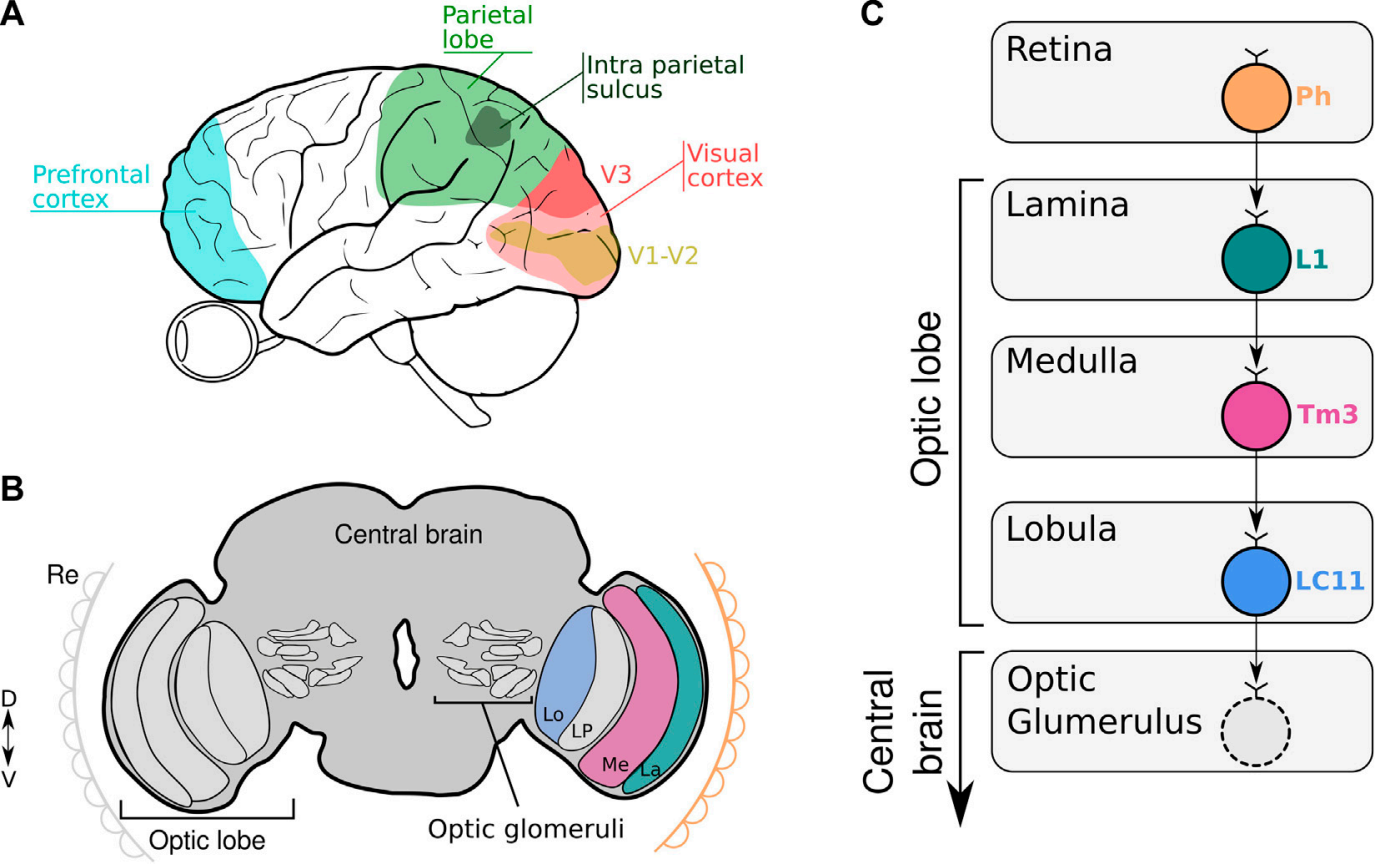
**(A, B)** The scheme illustrates the location of key areas of the human **(A)** and fruit fly brain **(B)** involved in numerical codification. **(A)** Numerical information is captured by the eye and process in the visual cortex, parietal lobe and prefrontal cortex. **(B)** Numerical information is captured by the retina (Re) and processed in the subsequent optic neuropiles (La: Lamina; Me: Medulla, Lo: Lobula and LP: Lobula Plate) to finally reach the central brain where the optic glomeruli are located. **(C)** Schematic diagram of neural circuits related to LC11 connectivity from the retina down to the different neuropiles within the optic lobe. Visual information is captured by the photoreceptors (Ph) in the retina and transferred to L1 neurons in the lamina. The information is transmitted to the Tm3 neurons in the medulla. In the lobula, LC11 neurons collect information from the Tm3 neurons. The optic glomerulus in the central brain receives inputs from LC11 neurons where the signals are transmitted to descending neurons leading to visually-guided behavior.

Humans’ number sense is the ability that allows us to represent and operate numerical quantities. The prefrontal cortex and the parietal lobe, specifically the intraparietal sulcus, have long been studied as the prime sources of numerical competence (Dehaene et al., 1999; Piazza et al., 2002; Castelli et al., 2006; Piazza et al., 2007). Examinations of brain-damaged patients and brain imaging studies have identified regions primarily in the posterior parietal and frontal lobes as key areas of number processing (Nieder and Dehaene, 2009; Figure 1A). Complementing these findings in humans, neurophysiological studies in monkeys have deciphered neuronal principles of numerical competence down to single neurons (Nieder, 2016). These studies show that numerosity-selective neurons in the prefrontal cortex and intraparietal sulcus exhibit maximum responses to the “preferred numerosity” (e.g. a specific number of items on a visual display) (Nieder et al., 2002). Moreover, single cell evidence for numerosity spontaneous selectivity were also shown in other animal models like crows (Wagener et al., 2018) and neonate domestic chicks (Kobylkov et al., 2022). This last study suggests that numerosity perception is possibly an inborn feature of the vertebrate brain.

In contrast, the contribution of subcortical areas is less studied due to the established concept that numerical cognition is highly abstract implying the involvement of associative and “higher-order” neural mechanisms located in the pallial territory of vertebrate animals. Nevertheless, 48 h old babies were able to discriminate numbers when the ratio was 3:1 or larger (Izard et al., 2009). This finding suggests that immature brains are able to detect and respond to numerosity. This “innate” faculty argues that numerosity could intuitively be perceived as a basic attribute of hard-wired sensory brain processes. Accordingly, Burr and Ross (2008) showed that numerosity is strongly susceptible to visual adaptation. Adult humans exposed to a given numerosity stimulus (e.g., dense or sparse dot clouds) for several seconds change the perception of subsequent stimuli. Observers who adapted to a large number of dots underestimated the number of dots on the new display, while observers who had adapted to a small number of dots overestimated the number of dots on the new display. This adaptation only occurred when the test sequence was displayed at the same position as the adaptor sequence (Burr and Ross, 2008). Therefore, it is possible to simultaneously adapt different locations of the visual field to high, low or neutral numerosities (Aagten-Murphy and Burr, 2016). Since adaptation is a characteristic of sense organs, numbers may be regarded as a sensory-like attribute. Another study found that tuning of human neural populations decreased during low and increased during high numerosity adaptation, indicating that preferred numerosities were predominantly attracted to the numerosity of the adapter (Tsouli et al., 2021). Recent reports showed that it is possible to decode the number of items seen by the subjects from the fMRI activity patterns in visual cortex areas (Bulthé et al., 2014; Bulthé et al., 2015; Eger et al., 2015; DeWind et al., 2019). With the advance in brain imaging technology, Castaldi et al. (2019) were able to split the fMRI signals into components specific to numbers and components corresponding to other related visual cues (e.g., density or size). These findings reveal a sensory extraction mechanism yielding information on numerosity separable from other dimensions already at visual stages (V1-V3) and suggest that the regions along the dorsal stream are most important for explicit manipulation of numerical quantity (Figure 1A). By using a computational modeling of human 7T fMRI data, Paul et al. (2022) recently demonstrated that numerosity estimation in humans could arise from the visual image representation at the level of primary visual cortex (V1). Item size and spacing have little effect on the numerical estimation in the spatial frequency domain, showing that numerosity itself could be accurately estimated very quickly in the brain. These studies strongly suggest a sensory processing mechanism capable of exploiting signals related to visual numerosity in humans.

## Numerical computation in fruit flies

Although numerical sensitivity has been shown in many invertebrates (Bortot et al., 2021), how the complex processing of numerosity is integrated within the structures of the brain to permit number-based visual discrimination remains unknown. This lack of neuronal evidence is due to the experimental difficulty of studying brain circuits associated with this cognitive capacity (Giurfa, 2019). The insect brain -although far from being simple- offers the best chance to understand the circuits involved in numerical processing at a neuronal level in a brain that is radically different from a vertebrate one.

Recently, we showed that *D. melanogaster* can discriminate between sets of objects based on numerosity [(Bengochea et al., 2022)- preprint, bioRxiv]. Flies consistently show a spontaneous preference for (*i.e.,* spend more time near) the larger set of objects, independently of the shape, size or the overall area of the set. Consistently with other animal models (Carazo et al., 2009; d’Ettorre et al., 2021), flies use numerical ratio to determine which set is larger. Importantly, flies -that innately prefer the larger numerosity- are able to associate the lower numerosity to a reward and to change their preference accordingly.

The visual system of *Drosophila* contains approximately 60,000 neurons that are organized in parallel, retinotopically arranged columns (Borst, 2009). In the visual system, the vertebrate retina and insect optic lobe share many anatomic and functional features in encoding visual information retinotopically (Sanes and Zipursky, 2010; Borst and Helmstaedter, 2015). Information tends to flow sequentially through different layers of neuropil that converge onto assemblies of columnar neurons in the lobula, the axons of which segregate to project to discrete optic glomeruli in the central brain (Figure 1B). Particularly, the third optic neuropil, the lobula, is a key structure in the brain of the fly involved in processing and extracting behaviorally relevant features from an animal’s environment. This is a neuropil that comprises many palisades of lobula columnar neurons (Cajal and Sánchez, 1915). Many of these LCs neurons have been characterized in great anatomical detail. For example, the LCs visual projection neurons of flies, similar to retinal ganglion cells in vertebrates, have been hypothesized to encode behaviorally relevant local visual objects dedicated to detection, such as other flies or predators (Strausfeld et al., 2007; Wu et al., 2016). Particularly, LC11 neurons are required for small object induced freezing behavior and have been shown to selectively respond to the movement of small targets in the visual field and weakly to bar-shaped objects (Keleş and Frye, 2017; Tanaka and Clark, 2020). These cells are key for sensing the movement of nearby conspecifics in a group and adjusting defensive behavior (Ferreira and Moita, 2020). We showed that silencing LC11 neurons caused a reduction in the spontaneous numerical discrimination abilities of flies. The silencing effect is also ratio dependent, observing the lack of discrimination with numerical ratio greater or equal to 0.50. Moreover, silencing a different type of lobula columnar neuron (LC10a) -which like LC11 also responds to small objects (Ribeiro et al., 2018; Hindmarsh Sten et al., 2021), do not prevent flies to show a preference for the larger numerosity. Importantly, silencing a high-order brain area -like the central complex- involved in visual navigation (Pfeiffer and Homberg, 2013; Seelig and Jayaraman, 2015) leaves the spontaneous numerical preference intact. This suggests not only some level of specificity for the role of LC11 in numerical processing, but also the rapid emergence of numerical discrimination in the visual system at the level of the optic lobes [(Bengochea et al., 2022)- preprint, bioRxiv].

The study of numerical abilities in invertebrates allows researchers to explore this cognitive property in animal models that have remarkably small brains but nonetheless reach cognitive solutions as those discovered by artificial neural networks. Moreover, models comprising as few as one (Rapp et al., 2020) or four neurons (Vasas and Chittka, 2019) demonstrate the capability of solving numerical tasks similar to insects. Vasas and Chittka (2019) developed a model which offers a non-countable magnitude estimation in which the success of discrimination is ratio dependent . Although we do not know whether flies sequentially count the objects or not, this model fits evidence of a ratio dependency in spontaneous and learned number discrimination in fruit flies. This model relies on inputs from the color-blind motion pathway and, therefore, is expected to use only long-wavelength-sensitive receptor inputs. Visual number stimuli are first detected by an on-off narrow-field phasic “brightness neuron” of the second optic lobe. Then, information arrives to a second neuron, the “brightness working memory neuron” which senses and responds maximally to changes in light intensity. In parallel, the brightness neuron sends a weaker input to a “counting working memory neuron” that responds proportionally to each change in brightness between dark and bright areas. All the information is finally collected by a fourth neuron that accumulates and provides continuous updates of the numerosity stimulus.

Accordingly, the literature regarding LC11 connectivity shows that LC11 neurons collect visual information from the ON visual pathway. It has been shown that the dendrites of LC11 cells receive inputs from transmedullary neuron 3 (Tm3) *via* GABAergic synapses (Takemura et al., 2011; Davis et al., 2020). Tm3 connects the proximal medulla and distal lobula. Like the brightness working memory neuron, Tm3 is an ON-OFF cell that responds maximally to any luminance change regardless of its polarity, and is tightly tuned to small moving objects (Keleş et al., 2020; Tanaka and Clark, 2020). Tm3 neurons collect the output of L1 neurons from the first neuropil which are the major input to the ON visual pathway. As the brightness neuron, L1 are ON-OFF phasic neurons that respond to each change in luminosity (Figure 1C). It has been shown that the physiological properties of the LC11 neurons fit well with a displacement detector (DD) model where they collect across the visual field the output of several size-tuned and fast-adapting cells (Tanaka and Clark, 2020). Furthermore, the spatial pooling in the DD model conceptually parallels how complex cells in mammalian visual cortex (V1) achieve phase invariance by pooling simple cell outputs (Movshon et al., 1978). Despite this being a plausible model, other neuronal configurations remain possible. One feasible model could involve a collector neuron downstream LC11 cells that senses the signaling of each LC11 neuron to a single object reaching its receptive field. Like this, numerical information would be encoded by the collector neuron that would fire only when more than one LC11 neuron is active. More experiments should be done in the feature to investigate the neuronal circuits and mechanisms encoding visual numerosity in invertebrates.

In summary, numerical discrimination in *Drosophila* depends on a cluster of visual neurons (LC11s) located in the optic lobe that are three synapses away from the retina, suggesting that for flies “numbers” are attributes of the visual scene and thus numerosity is processed at a sensory level.

## Conclusion

Numerosity estimation is fundamental to animal survival. In terms of how humans compute numerical information, there is a clear distinction between the spatial selectivity of visual monotonically responding populations at the level of V1 and tuned populations in association cortices. Numerosity perception also mirrors to a certain degree sensory activity and its susceptibility to adaptation suggests that numerosity is a visual property (Burr and Ross, 2008; Burr et al., 2018). Non-etheless, the nature of the neuronal computations underlying this “visual sense of number” remains controversial (Durgin, 2008). This is mainly because researchers have not been able to conclusively demonstrate that numerosity-tuned responses at the level of visual cortex V1-V3 are derived from early visual frequency domain image representations, since it is not possible to physiologically disrupt the visual image representation and show effects on numerosity-tuned responses. In this sense, studying visual number representations in animal models that allow brain manipulation will shed light on the neuronal mechanism of visual processing of numbers. Part of the neural network associated with the estimation of continuous and discrete quantity in the zebrafish brain has been identified (Messina et al., 2020). Recent studies showed that a particular area of the telencephalon (*dorsalis telencephali*) responds selectively to numerosity (Messina et al., 2021). However, these discoveries were found *a posteriori* of the numerical task by detecting high levels of *c-fos* expression (e.g. neuronal activity) in those areas.

In mammals, multiple regions of the brain, for example the parietal cortex and the prefrontal cortex, are involved in the processing of numerosity (review in (Lorenzi et al., 2021)). We hypothesize that it will be also the case in fruit flies. It may be necessary to integrate multiple neuronal responses to generate a more reliable estimation of numerosity. It would be unlikely that only one type of visual neuron would be required for numerical performance, however it remains to test other visual neuron types and other areas in the central brain. To be able to understand the brain areas and neuronal circuitry involved in numerosity it is necessary to record (e.g., functional imaging) and manipulate those particular areas while the animals are performing the numerical task (Messina et al., 2022). In this regard, *Drosophila* offers several advantages. Flies have not only been shown to have a spontaneous preference for larger numerosities, but also demonstrate that they can associate a specific numerosity to a reward and change their preference accordingly [(Bengochea et al., 2022)- preprint, bioRxiv]. The genetic tools that are available will allow us to disentangle the interaction of multiple brain regions, neurons and circuits by manipulating them while the flies are making their numerical decision. We propose the fruit fly as an advantageous model to study, not only the brain areas involved in numerical processing, but also brain areas related to the change in preference after learning. Future studies will provide important insights into the basic neuronal mechanism underlying numerosity processing.

## Data Availability

The original contributions presented in the study are included in the article/supplementary material, further inquiries can be directed to the corresponding authors.
